# Supplementary material for: Slc26a1 is not essential for spermatogenesis and male fertility in mice
Source: PeerJ. 2023 Dec 15;11:e16558. doi: 10.7717/peerj.16558 (PMC10726749; doi:10.7717/peerj.16558)
Supplement: Supplemental Information 1 — Slc26a, solute carrier 26A [file peerj-11-16558-s001.docx]

**Table S1**   **Primer Sequence**

| Gene | Forward | Reverse | Product length |
| --- | --- | --- | --- |
| Slc26a1  Slc26a2  Slc26a3  Slc26a4  Slc26a5  Slc26a6  Slc26a7  Slc26a8  Slc26a9  Slc26a10  Slc26a11  18s RNA | AGCACTTGGCATAATGGGCA  TTGGTACATCCCGCCACATC  GGAGGCAAAACACAGGTTGC  CTCAGGGCAACCAAGAACGG  CAGCTTCCCCAAGGTCCTTTT  AACTTGGTTCCGATGCTCCC  TGCTCCCCCAATGAACATCC  GCCACCGGAAACCAGCTAAT  TCTGGTAAACATCCACGCCC  CAGAGAACCTCTCACCCACG  TAGGCCCAAGACTCAGGTGT  AAACGGCTACCACATCCAAG | AGGCTCTTTGGCTTATCTGTTCT  AAAGAAGCCCATTGCTACCTGA  GGCCTAATCCGAGTCCAAGG  GGAAGGATACAGCTCCGCAA  GCATCTCTGGCTTCTGTCCC  CTGCCCACCATCACAGACAT  TTAGACAAGCCACCTGCGTC  CGTGGTGCTTGAAAGTGGTG  TGGTCCCGAACATCTCCTGT  GGGCAAAAGCCATGCCCTG  CCCACGATCACGGTGTAGTC  CCTCCAATGGATCCTCGTTA | 178  257  244  214  128  297  260  201  248  278  186  155 |

Slc26a, solute carrier 26A
