# Supplementary material for: Slc26a1 is not essential for spermatogenesis and male fertility in mice
Source: PeerJ. 2023 Dec 15;11:e16558. doi: 10.7717/peerj.16558 (PMC10726749; doi:10.7717/peerj.16558)
Supplement: Supplemental Information 2 — SLC26A1, solute carrier 26A; Lin28, lin-28 homolog; SOX9, SRY-box 9; SCP3, synaptonemal complex protein 3; γH2AX, H2AX variant histone; 3β-HSD, IgG3 κ mouse monoclonal 3 beta-HSD. [file peerj-11-16558-s002.docx]

**Table S2**  Antibodies

| Antigen | Source | Company | Application | Dilution | PRODUCT NUMBER |
| --- | --- | --- | --- | --- | --- |
| SLC26A1  Lin28  SOX9  γH2AX  3β-HSD  Tubulin | Rabbit  Rabbit  Rabbit Mouse  Goat  Mouse | Novus Abcam  Millipore Abcam  Santa Cruze  Beytime | IF;WB  IF  IF  IF  IF  WB | 1:1000  1:200  1:400  1:400  1:200  1:10000 | NBP1-84897  AB46020  AB5535  AB26350  SC-515120  AT819 |

SLC26A1, solute carrier 26A; Lin28, lin-28 homolog; SOX9, SRY-box 9; SCP3, synaptonemal complex protein 3; γH2AX, H2AX variant histone; 3β-HSD, IgG3 κ mouse monoclonal 3 beta-HSD.
