# Supplementary figures and images for: Slc26a1 is not essential for spermatogenesis and male fertility in mice
Source: PeerJ. 2023 Dec 15;11:e16558. doi: 10.7717/peerj.16558 (PMC10726749; doi:10.7717/peerj.16558)

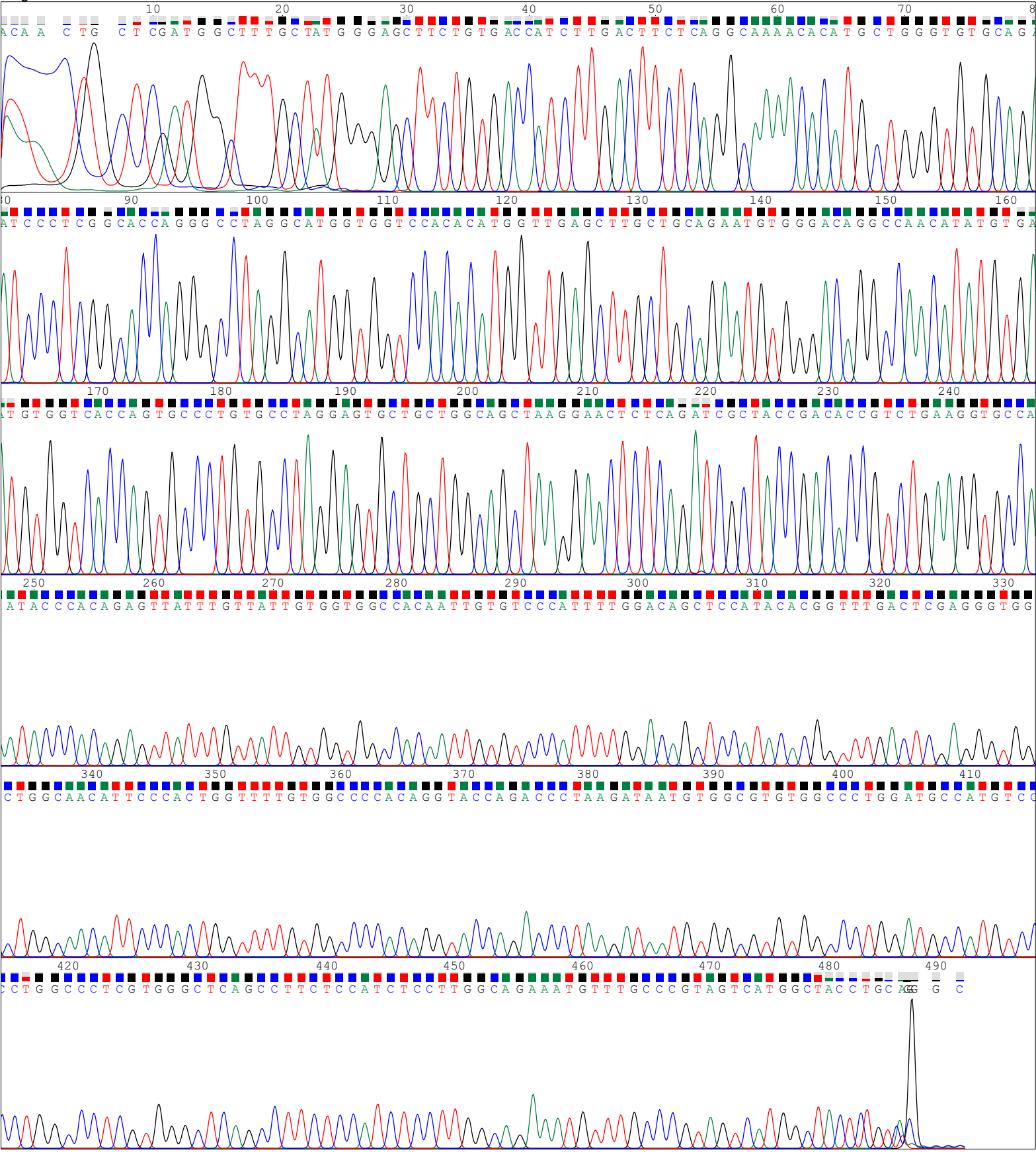

Supplement: Supplemental Information 5 [file peerj-11-16558-s005.pdf]

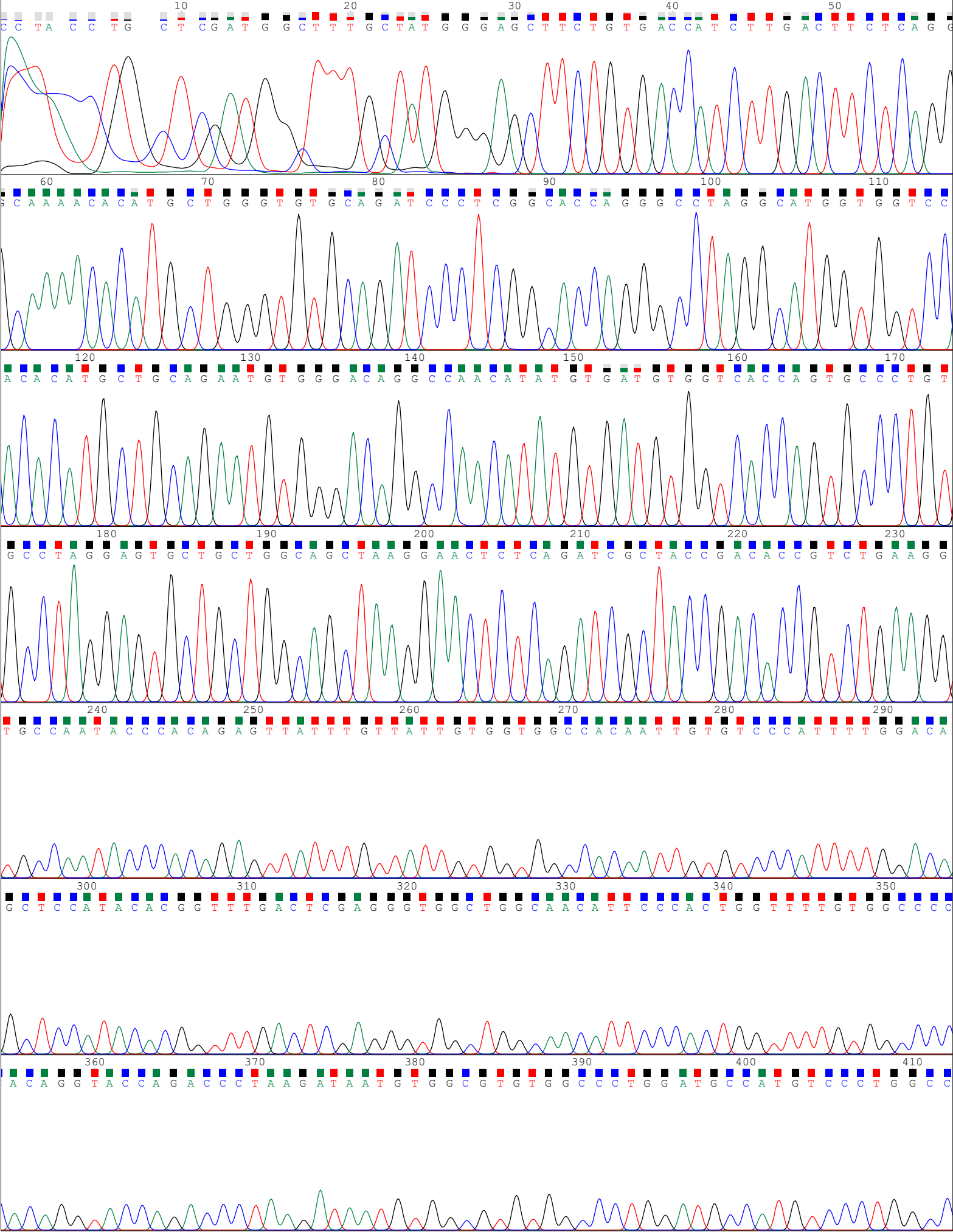

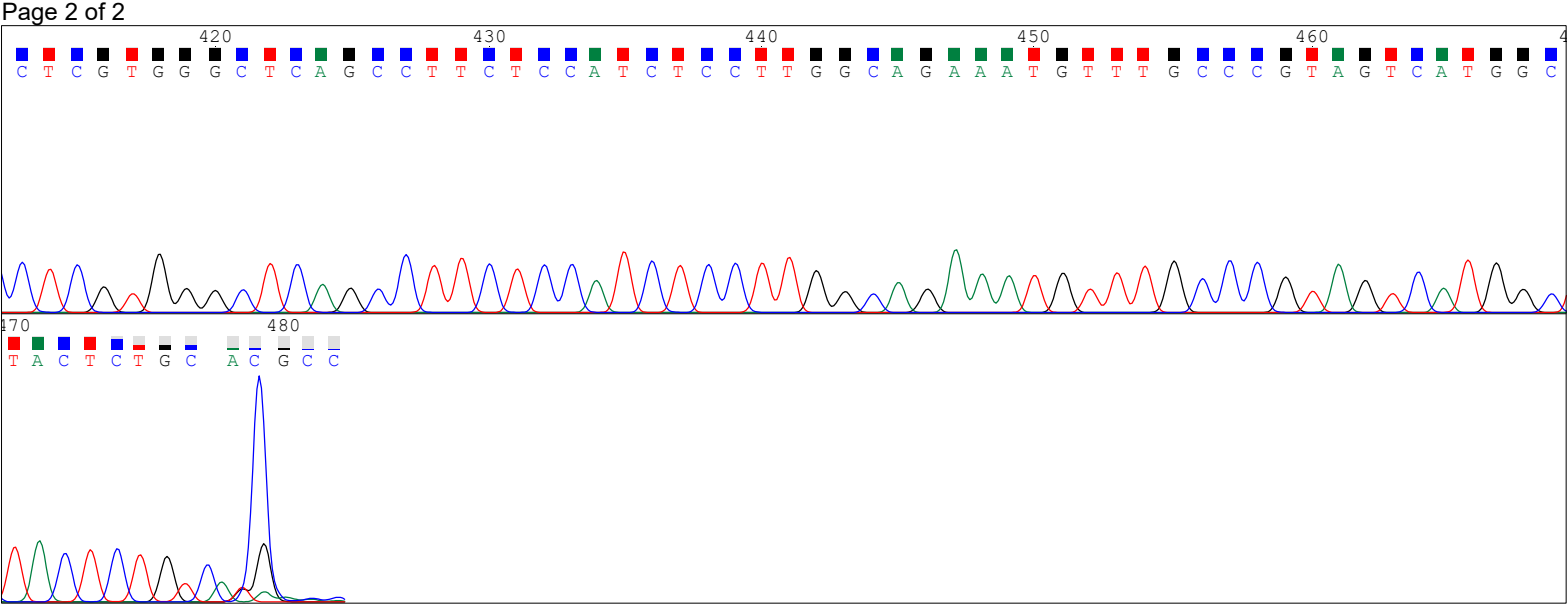

Supplement: Supplemental Information 7 [file peerj-11-16558-s007.pdf]
